# Murine mammary tumor cells with a claudin-low genotype

**DOI:** 10.1186/1475-2867-11-28

**Published:** 2011-08-16

**Authors:** Craig I Campbell, Devan E Thompson, Megan D Siwicky, Roger A Moorehead

**Affiliations:** 1 Department of Biomedical Sciences, Ontario Veterinary College, University of Guelph, Guelph, ON N1G2W1, Canada; 2 Department of Biomedical Sciences, Ontario Veterinary College, University of Guelph, 50 Stone Road East, Guelph, ON N5A7Z1, Canada

## Abstract

**Background:**

Molecular classification of human breast cancers has identified at least 5 distinct tumor subtypes; luminal A, luminal B, Her2-enriched, basal-like and claudin-low. The claudin-low subtype was identified in 2007 and is characterized by low expression of luminal differentiation markers and claudins 3, 4 and 7 and high levels of mesenchymal markers. Claudin-low tumors have a reported prevalence of 7-14% and these tumors have a poor prognosis.

**Results:**

In this study we report the characterization of several cell lines established from mammary tumors that develop in MTB-IGFIR transgenic mice. Two lines, RM11A and RJ348 present with histological features and gene expression patterns that resemble claudin-low breast tumors. Specifically, RM11A and RJ348 cells express high levels of the mesenchymal genes Zeb1, Zeb2, Twist1 and Twist2 and very low levels of E-cadherin and claudins 3, 4 and 7. The RM11A and RJ348 cells are also highly tumorigenic when re-introduced into the mammary fat pad of mice.

**Conclusions:**

Mammary tumor cells established from MTB-IGFIR transgenic mice can be used as in vitro and in vivo model systems to further our understanding of the poorly characterized, claudin-low, breast cancer subtype.

## Background

Molecular profiling has identified 5 distinct subtypes of human breast tumors, luminal A, luminal B, HER2-enriched, basal-like and claudin-low [1-7]. Luminal A and luminal B tumors are estrogen receptor positive, cytokeratin 8 and 18 positive and have a good prognosis. Luminal B tumors have similar characteristics as luminal A tumors but have a poorer prognosis than luminal A tumors [1-3]. HER2-enriched tumors are characterized by high expression of HER2 while basal-like tumors do not express cytokeratins associated with luminal epithelial cells (cytokeratins 8 and 18) but do express cytokeratins 5, 6 and 14 as well as vimentin [1-3]. Claudin-low tumors express mesenchymal genes such as Twist1, Twist2, Zeb1, Zeb2, Slug and Snail and low levels of E-cadherin and claudins 3, 4 and 7 [6,7].

One approach to determining which subtype of human breast cancer a particular mouse mammary tumor or mammary tumor cell line is in fact modeling is through the use of DNA microarrays and cluster analysis with human tumors. We utilized this approach for the mammary tumors that arise in the MTB-IGFIR transgenic mice. Overexpression of IGF-IR in mammary epithelial cells in MTB-IGFIR transgenic mice leads to the development of mammary tumors and these tumors have been designated primary mammary tumors or PMTs [8]. Downregulation of the IGF-IR transgene in established mammary tumors results in the regression of most of the tumors, however, some of the tumors recur and do not express the IGF-IR transgene. Since these tumors that recur take on a spindle-shape morphology they have been designated recurrent spindle tumors or RSTs [9].

Cluster analysis with human breast tumors revealed that the PMT samples cluster most closely with 51 of 58 human basal-like tumors and a small number of claudin-low and HER2-enriched tumors. The RSTs clustered with a group of 5 claudin-low breast tumors and 1 normal sample (unpublished observation).

In this manuscript, 3 cell lines derived from MTB-IGFIR mammary tumors have been characterized. It was observed that two of these cell lines, RM11A and RJ348, express mesenchymal genes, particularly Twist1, Twist2, Zeb1, Zeb2 and vimentin and low levels of E-cadherin as well as claudins 3, 4 and 7 indicating that these cells most likely resemble human claudin-low tumors. One other cell line, RJ345 retains the expression of claudins 3, 4 and 7, E-cadherin and expresses only low levels of vimentin. Based on this gene expression pattern, RJ345 cells most likely resemble human luminal tumors. Importantly, the RM11A and RJ348 cells rapidly form tumors when re-introduced into the mammary fad pads of wild type mice thus providing an in vivo model to complement the in vitro studies.

## Materials and methods

### Cell lines and culture conditions

Cell lines used for this study were derived from tumors that developed in MTB-IGFIR transgenic mice as described in [8]. RM11A cells were isolated from a primary mammary tumor that expressed high levels of IGF-IR transgene and have been previously characterized [8,10]. RJ345 cells were isolated from an independent MTB-IGFIR mammary tumor that expressed high levels of IGF-IR. RJ348 cells were isolated from an IGF-IR-independent recurrent tumor [9]. RM11A, RJ345 and RJ348 cells were cultured in media described in [10] with RM11A and RJ345 being cultured in media supplemented with 10 μg/mL doxycycline for transgene induction while RJ348 cells were cultured in doxycycline-free media. 4T1 cells, characterized in [11] were obtained from the ATCC (Manassas, VA) and were cultured in DMEM media containing 10% FBS, 1 mM sodium pyruvate, 10 mM Hepes and 4 mM L-glutamine. MCF-7 cells were cultured in DMEM media containing 10% FBS and 4 mM L-glutamine while MDA-MB-231 cells were cultured in RPMI media containing 5% FBS and 4 mM L-glutamine. All cells were maintained at 37°C with 5% CO_2_.

### Real time PCR

Total RNA was extracted from cells cultured to approximately 75% confluency, 2d after plating using the Ambion *mir*VANA miRNA isolation kit (Applied Biosystems, Streetsville, ON), in accordance with the manufacturer's instructions (without the miRNA enrichment step). Real time PCR was performed as described in [9]. All primers were obtained from Origene (Rockville, MD).

### Animal experiments

Syngeneic mammary gland injections were performed as described in [10]. Briefly, 1 × 10^3^, 1 × 10^4^, or 5 × 10^5 ^RJ348 cells or 5 × 10^5 ^or 2.5 × 10^6 ^RJ345 cells were injected in each 4^th ^inguinal mammary gland of wild type FVB mice. Mice were palpated twice per week. Metastasis was assessed through serial sectioning of lung tissue with subsequent H&E staining. Animals were housed and cared for following the guidelines established by the Central Animal Facility at the University of Guelph and the Canadian Council on Animal Care.

### Statistics

Statistical significance was determined using an ANOVA followed by a Tukey's HSD post-hoc test. Values were considered statistically significant when p < 0.05.

## Results

Previous work demonstrated that IGF-IR overexpression in mammary epithelial cells in MTB-IGFIR transgenic mice resulted in the development of mammary tumors with characteristics similar to human basal breast tumors (unpublished observations). Immunohistochemical analysis revealed that these tumors contained cells expressing a mixture of luminal and basal cytokeratins [8]. The mammary tumors that develop following IGF-IR downregulation have characteristics similar to human claudin-low tumors. Immunohistochemical analysis of these tumors revealed that these tumor cells expressed primarily basal cytokeratins [9]. To facilitate our understanding of this model, 3 cell lines have been derived. Two of cell lines (RJ345 and RM11A) were derived from primary mammary tumor that expressed high levels of IGF-IR while RJ348 was derived from a tumor that recurred following IGF-IR downregulation (Table 1).

**Table 1:** Source of Mammary Tumor Cell Lines

Cell Line	Tumor Derived From	Human Breast Tumor Similarity^1^
RJ345	primary mammary tumor	51/58 basal-like, 4/17 claudin-low and 2/31 HER2-enriched tumors

RM11A	primary mammary tumor	51/58 basal-like, 4/17 claudin-low and 2/31 HER2-enriched tumors

RJ348	recurrent spindle tumor	5/17 human claudin-low tumors and 1/15 normal samples

^1^Human breast tumor similarity is based on cluster analysis of our primary mammary tumors and recurrent spindle tumors with human breast tumor samples as described in [6].

To characterize the murine mammary tumor cell lines derived from MTB-IGFIR transgenic mice [8,11], qRT-PCR was performed for the epithelial marker E-cadherin and several mesenchymal genes. As a control, 4T1 cells were included in this experiment as they have previously been shown to express high levels of E-cadherin [12]. As shown in Table 2, 4T1 and RJ345 cells express the highest levels of E-cadherin while RJ348 and RM11A cells express very low levels of E-cadherin. With respect to mesenchymal genes, RJ348 and RM11A cells expressed higher levels of Twist1, Twist2, Zeb1, Zeb2 than the 4T1 and RJ345 cells (Table 2).

**Table 2:** Expression of Epithelial and Mesenchymal Genes

Gene	4T1	RJ345	RJ348	RM11A
E-cadherin	154 ± 28.2	75.0 ± 19.8	0.87 ± 0.15^a^	1.01 ± 0.06^a^

Twist1	0.29 ± 0.05	0.13 ± 0.02	0.67 ± 0.06	0.65 ± 0.23

Twist2	0.02 ± 0.01	0.13 ± 0.04	0.90 ± 0.15^a, b^	0.91 ± 0.06^a, b^

Zeb1	0.02 ± 0.01	0.12 ± 0.04	0.89 ± 0.22^a, b^	1.50 ± 0.16^a, b^

Zeb2	0.31 ± 0.09	0.02 ± 0.01	0.95 ± 0.04^a, b^	1.08 ± 0.18^a, b^

Data is expressed relative to HPRT. Values are displayed as mean ± SEM; n = 3

^a^Significantly different (p < 0.05) than 4T1 cells

^b^Significantly different (p < 0.05) than RJ345 cells.

**Figure 1:**
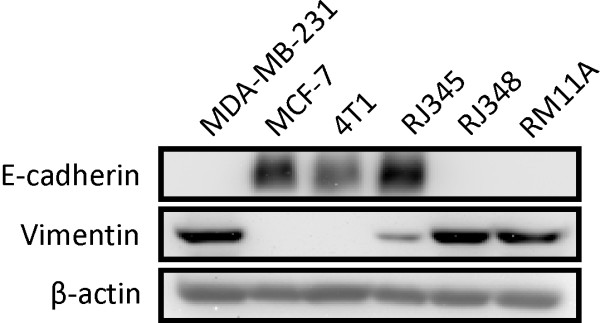
**Western blot of the epithelial marker E-cadherin and the mesenchymal marker vimentin in 4T1, RJ345, RJ48, RM11A, MCF-7 and MDA-MB-231 cells**. β-actin was used as a loading control.

Next western blotting was used to confirm the high level of E-cadherin protein in the 4T1 and RJ345 cells (Figure 1). In addition, vimentin, a mesenchymal marker, was found at high levels in the RJ348 cells and RM11A cells and at lower levels in the 4T1 and RJ345 cells. Two human breast cancer cell lines, MCF-7 which represent human luminal tumors and MDA-MB-231 which represent human claudin-low tumors were included as controls in the western blots. As expected, MCF-7 cells expressed high levels of E-cadherin and essentially no vimentin while MDA-MB-231 had the opposite pattern of expression (Figure 1).

Since the tumors that recurred following IGF-IR downregulation clustered with claudin-low tumors, the expression of claudins 3, 4 and 7 were evaluated in the murine mammary tumor cell lines. RT-PCR analysis revealed that RJ348 and RM11A cells had considerably lower levels of claudins 3, 4 and 7 compared to either the 4T1 or RJ345 cells (Table 3).

**Table 3:** Expression of Claudin Genes

Gene	4T1	RJ345	RJ348	RM11A
Claudin-3	95.8 ± 73.8	25.2 ± 13.8	0.49 ± 0.23	0.72 ± 0.39

Claudin-4	520 ± 56.3	252 ± 74.5	0.31 ± 0.14^a,b^	0.53 ± 0.26^a,b^

Claudin-7	167 ± 27.7	99.5 ± 31.6	0.20 ± 0.08^a,b^	0.47 ± 0.27^a,b^

Data is expressed relative to HPRT. Values are displayed as mean ± SEM.

^a^Significantly different (p < 0.05) than 4T1 cells

^b^Significantly different (p < 0.05) than RJ345 cells.

The ability of 4T1 [11] and RM11A [10] cells to form tumors when injected into the mammary fat pad of syngeneic mice has previously been described. Since the ability of RJ345 and RJ438 cells to form tumors following intramammary injection had not been evaluated, cells from these lines were injected into the mammary fat pads of wild type, female FVB mice. The initial round of injections utilized 5 × 10^5 ^cells for each of the cell lines, RJ345 and RJ348. By 11 days post injection, all of the mice receiving 5 × 10^5 ^RJ348 cells had palpable tumors in both mammary glands (Table 4). By 20 days post injection the tumors had reached approximately 17 mm in diameter (the maximum size allowed by the Canadian Council of Animal Care) and mice were therefore euthanized. RJ345 cells failed to form tumors even after 14 weeks (the point at which the experiment was ended). Based on these findings, a second round of injections were performed using either 1 × 10^3 ^or 1 × 10^4 ^RJ348 cells or 2.5 × 10^6 ^RJ345 cells. Both concentrations of the RJ348 injections produced tumors however the tumor incidence declined to 33% when only 1 × 10^3 ^RJ348 cells were injected. One observation with the injection of 1 × 10^3 ^RJ348 cells is that the tumors frequently became ulcerated when tumors were only approximately 7 mm in diameter. RJ345 cells formed tumors when 2.5 × 10^6 ^cells were utilized in 50% of the mammary glands. These tumors were not palpable 14 weeks after injection but were found during necropsy.

**Table 4:** Tumor Incidence Following Injection of RJ345 or RJ348 cells

Cell Line	Number of Cells Injected	Number of Mammary Glands Injected	Number of Mammary Glands with Tumors (percentage)	Tumor Onset (days)
RJ345	5 × 10^5^	14	0 (0%)	N/A^1^

RJ345	2.5 × 10^6^	6	3 (50%)	N/A^1^

RJ348	1 × 10^3^	10	3 (33%)	43.6 ± 17.7^1,2^

RJ348	1 × 10^4^	10	10 (100%)	22.7 ± 2.0

RJ348	5 × 10^5^	10	10 (100%)	11 ± 0

^1^Mice with non-palpable tumors were euthanized 14 weeks after tumor cell injection.

^2^Numbers represent mean ± SEM.

**Figure 2:**
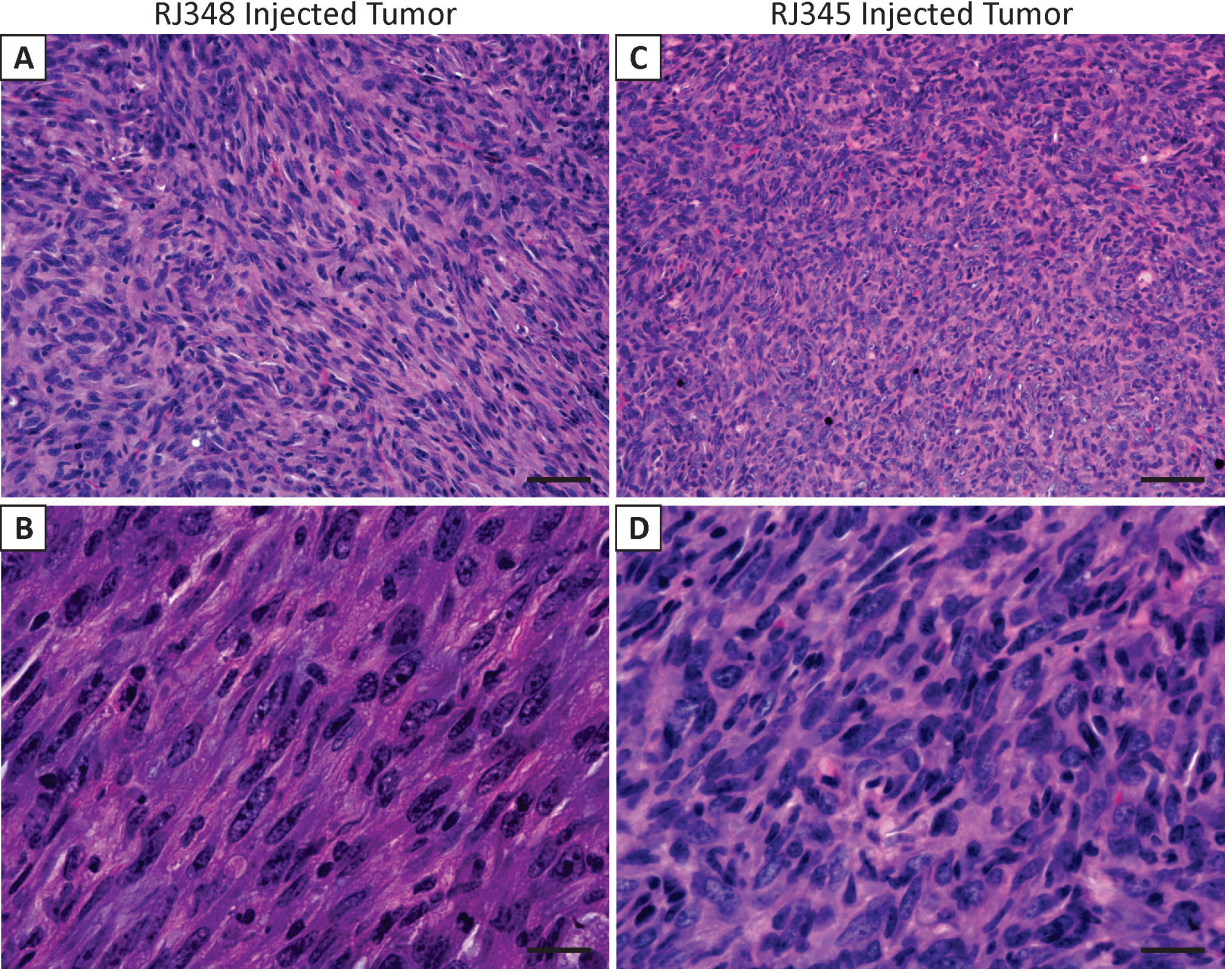
**Hematoxylin and Eosin stained sections of tumors induced by injection of RJ348 cells (A, B) or RJ345 cells (C, D) into the mammary fat pad of wild type mice**. Panels B and D represent higher magnification images of panels A and B, respectively. Scale bars 100 μm for A and C and 50 μm for B and D.

Histological analysis of the RJ348 mammary tumors revealed tumors with spindle shaped morphology (Figure 2A, B). The tumors induced by the injection of RJ345 cells produced tumors with similar histologic features to the RJ348 injections (Figure 2C, D). Lung tissue from tumor bearing mice was analyzed macroscopically and microscopically for the presence of metastasis; no such lesions were identified (data not shown).

## Discussion

We have recently performed DNA microarray analysis and hierarchal clustering of mammary tumors that develop in MTB-IGFIR transgenic mice with other mouse mammary tumor models and with human breast tumors. It was observed that the mammary tumors induced by IGF-IR overexpression clustered most closely with murine mammary tumor models presenting with a basal or mixed cell lineage tumors and primarily with human basal breast tumors. The mammary tumors of the MTB-IGFIR transgenic mice that recur following IGF-IR downregulation cluster most closely with murine mammary tumor models presenting with basal/myoepithelial features and primarily with human claudin-low tumors (unpublished observation).

To complement our transgenic approach, cell lines have been isolated from some of the mammary tumors that arose in the MTB-IGFIR transgenic mice. One cell line, RM11A, has been partially characterized previously and this cell line displayed a spindle-shaped morphology in culture and expressed basal cytokeratins (cytokeratins 5 and 14) despite being derived from a tumor presenting with histologic features consistent with a luminal phenotype. These luminal appearing tumors do frequently contain clusters of cells that stain positive for basal cytokeratins and take on a more mesenchymal phenotype [9]. This study confirms the findings of the previous report [10] and further demonstrates that RM11A cells express the mesenchymal protein vimentin, a number of mesenchymal genes (Twist1, Twist2, Zeb1 and Zeb2) and little or no E-cadherin (luminal epithelial marker). In addition, the RM11A cells expressed low levels of claudins 3, 4 and 7 suggesting that these cells have a gene expression pattern similar with claudin-low tumors. Similarly, the RJ348 cells expressed a similar gene expression profile suggesting that they to represent claudin-low breast tumors.

These findings suggest that although a majority of the cells within mammary tumors induced by IGF-IR overexpression appear luminal, these tumors share gene expression profiles with human basal tumors. The fact that RM11A cells possess claudin-low features also suggests that some of the tumors may have small pockets of cells containing a claudin-low genotype. These small pockets of claudin-low cells or individual claudin-low cells may explain why all of the small mammary tumors and most of the large mammary tumors regress to a non-palpable state following IGF-IR downregulation [9]. Since claudin-low tumors possess features of stem cells [7], it is possible that the tumors that regress and recur or only partially regress are those that contain claudin-low cells. If RJ348 cells are enriched with stem cell characteristics, this would explain why as few as 1000 injected cells are capable if initiating mammary tumor development (similar limiting dilution studies have not been performed in RM11A cells).

Claudin-low tumors were first identified by Herschkowitz et al [6] as a distinct molecular subtype of human breast cancer. These tumors have a prevalence of approximately 7-14% and a have a poor prognosis compared to luminal A tumors [7]. Although a number of human tumor cell lines have been identified as sharing features of claudin-low tumors (MDA-MB-435, MDA-MB-436, Hs578T, BT549, MDA-MB-231, MDA-MB-157, SUM1315MO2, SUM159PT and HBL100), fewer claudin-low, murine mammary tumor cell lines have been established [7]. Since the RM11A and RJ348 cells form tumors when re-injected into the mammary fat pat, these cells provide us with a system where claudin-low tumors can be evaluated in vitro and in vivo in immunocompetent mice. The immune status of mice may be particularly important when studying claudin-low tumors as one of the hallmarks of this tumor type is an increase in immune-related genes [7].

RJ345 cells express the epithelial marker E-cadherin and little or no mesenchymal markers suggesting that these cells represent luminal or basal epithelial cells. The fact that RJ345 cells form mammary tumors only after the injection of 2.5 × 10^6 ^cells suggest that this cell line is weekly tumorigenic and this may be due to the small number of cells with stem cell characteristics. Since these cells are only weakly tumorigenic, they will be useful in identifying genes that facilitate tumorigenesis through gene alteration and subsequent mammary transplantation. The RJ345 cells will be useful in identifying genes that facilitate tumor initiation and growth.

Therefore, we have created a model system where tumor cells with claudin-low characteristics can be evaluated in vitro and in vivo. This system should improve our understanding of human claudin-low breast tumors and possible therapeutic strategies to treat these tumors which typically have a poor prognosis.

## Competing interests

The authors declare that they have no competing interests.

## Authors' contributions

CIC performed all of the real-time PCR, injected all the mice with tumor cells, monitored mice following the first round of tumor cell injections and collected tissue following the first round of tumor cell injections. DET completed the monitoring of mice and tissue collection following the second round of tumor cell injections, sectioned the tissue and performed H&E staining from the second round of tumor cell injections. MDS performed the western blot for E-cadherin and vimentin and performed the sectioning and staining of the tumor tissue from the first round of tumor cell injections. RAM conceived the study, coordinated the study and wrote the manuscript. All authors read and approved the final manuscript.
